# Photoelectrocatalytic degradation of high-density polyethylene microplastics on TiO_2_-modified boron-doped diamond photoanode

**DOI:** 10.1016/j.isci.2024.109192

**Published:** 2024-02-12

**Authors:** Wendy Quilumbaquin, G. Xavier Castillo-Cabrera, Luis J. Borrero-González, José R. Mora, Vladimir Valle, Alexis Debut, Luis D. Loor-Urgilés, Patricio J. Espinoza-Montero

**Affiliations:** 1 Escuela de Ciencias Químicas, Pontificia Universidad Católica del Ecuador, Quito 170525, Ecuador; 2 Laboratorio de Óptica Aplicada, Escuela de Ciencias Físicas y Matemática, Pontificia Universidad Católica del Ecuador, Quito 170525, Ecuador; 3 Department of Chemical Engineering, Universidad San Francisco de Quito USFQ, Quito 170157, Ecuador; 4 Departamento de Ciencias de Alimentos y Biotecnología, Escuela Politécnica Nacional, Quito 170517, Ecuador; 5 Centro de Nanociencia y Nanotecnología, Universidad de las Fuerzas Armadas ESPE, Sangolquí 170501, Ecuador

**Keywords:** Materials chemistry, Devices

## Abstract

Microplastic (MP) accumulation in the environment is accelerating rapidly, which has led to their effects on both the ecosystem and human life garnering much attention. This study is the first to examine the degradation of high-density polyethylene (HDPE) MPs via photoelectrocatalysis (PEC) using a TiO_2_-modified boron-doped diamond (BDD/TiO_2_) photoanode. This study was divided into three stages: (i) preparation of the photoanode through electrophoretic deposition of synthetic TiO_2_ nanoparticles on a BDD electrode; (ii) characterization of the modified photoanode using electrochemical, structural, and optical techniques; and (iii) degradation of HDPE MPs by electrochemical oxidation and photoelectrocatalysis on bare and modified BDD electrodes under dark and UV light conditions. The results indicate that the PEC technique degraded 89.91 ± 0.08% of HDPE MPs in a 10-h reaction and was more efficient at a lower current density (6.89 mA cm^−1^) with the BDD/TiO_2_ photoanode compared to electrochemical oxidation on bare BDD.

## Highlights

• The BDD/TiO_2_ photoanode was prepared by electrophoretic method
• BDD/TiO_2_ exhibited excellent photocurrent and charge transfer resistance reduction
• Superior degradation efficiency of HDPE-MPs was achieved by PEC compared to EO

## Graphical abstract

**Figure:**
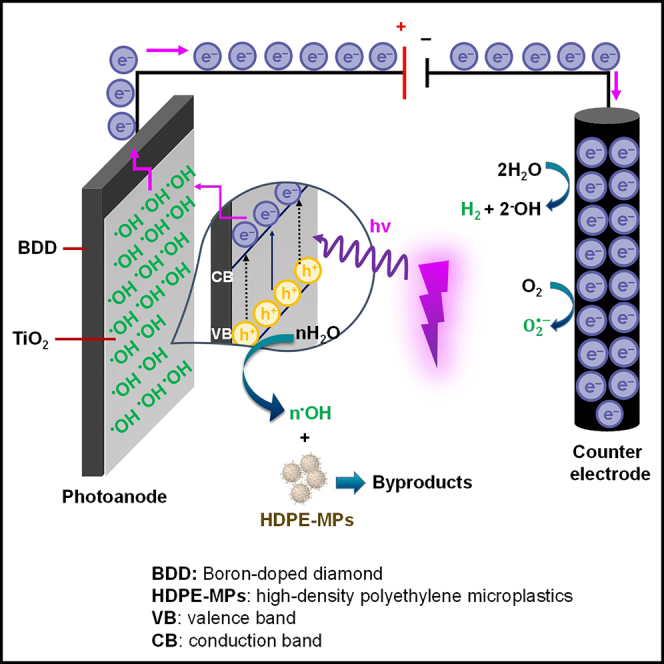

## Introduction

The amount of plastic products used reached almost 370 million tons in 2019.^1^ Perhaps more alarmingly, is that approximately 10 million tons of plastics are released into the oceans every year as pollution, and an estimated 2.7 × 10^5^ tons of plastic are currently floating in the ocean.^2^ Plastics begin to slowly degrade into microplastics (MPs) mainly as a result of the environmental conditions to which they are exposed (e.g., solar radiation, microorganisms, salinity, humidity, and abrasive effects).^2^ Researchers’ have taken steps to study MPs (<5 mm) as water source contaminants because synthetic polymers are considered hazardous waste, and thus studies should be extended to soils, biota, and the earth’s atmosphere.^3^^,^^4^ Polyethylene (PE) is indeed one of the most widely used commercial plastics in the world.^5^ Its versatility, durability, and low cost make it a popular choice for various applications, and it is also frequently found in marine environments.^6^^,^^7^ However, the monitoring of MP pollution has been so challenging that it has been necessary to create the concept of a “microplastic cycle.”^8^ In this cycle, MPs’ harmful effects consistently occur through ingestion at all trophic levels by both individual organisms and species assemblages.^9^^,^^10^^,^^11^ Specific research on MPs’ consequences for the health of humans and other organisms is in the early stages. For instance, Feckelman et al. demonstrated that current levels of MPs pollution are leaving certain seabirds susceptible to their ingestion, finding that MPs affect the gut biomes of these birds.^12^ In humans, MPs made from PE and polystyrene have been shown to induce cytotoxicity due to oxidative stress for both cerebral and epithelial (surface lining) human cells.^13^ Moreover, MPs enter the body via ingestion and inhalation, and their harmful effects are likely related to cellular damage and immune and inflammatory reactions.^14^ Despite such evidence, environmental remediation by catalysis is only a recent and minor effort in the face of a major environmental dilemma. Modern technologies adapted for removal or remediation is based on the application of physical, chemical, and biological processes or some combination. Of these, advanced oxidation processes (AOPs) should be an important focus.^15^^,^^16^^,^^17^^,^^18^

Previous research has shown that AOPs may be promising for the efficient removal of a wide variety of contaminants.^17^^,^^19^ Among the AOPs, photoelectrocatalysis (PEC) stands out as an environmentally friendly and efficient technology, as it strikes a balance between the energy costs of conventional heterogeneous photocatalysis and advanced electrochemical oxidation (EO).^20^^,^^21^ As a simple explanation, PEC can be divided into the following stages. First, light is absorbed on a crystal-lattice, photocatalytic thin film supported on a conductive substrate (photoelectrode) (light energy > photocatalyst band gap), generating electron-hole charge carriers ${(e}_{\text{CB}}^{–}/h_{\text{VB}}^{+})$. Second, $e_{\text{CB}}^{–}$ is extracted from the photoanode’s conduction band minima (CBM) through external circuit, applying an external bias potential to avoid recombination and making $h_{\text{VB}}^{+}$ available. Finally, $h_{\text{VB}}^{+}$-mediated redox reactions occur ($h_{\text{VB}}^{+}$ are expected to have sufficient energy to oxidize water to form hydroxyl radicals (^•^OH) or oxidize organic matter directly).^22^^,^^23^ The major disadvantage of traditional heterogeneous photocatalysis is the short lifetime of the charge carriers. After photoexcitation of electron-hole pairs—in the femtosecond regime—and without the application of direct biasing, the photoexcited carriers tend to recombine at ultrafast rates, although slower than the excitation, which greatly competes with the charge transfer and thus with catalysis.^24^ Furthermore, the recovery efficiency of the solid-state photocatalyst is limited for most applications.^25^ On the other hand, and despite the advantages of PEC, its overall efficiency is not without experimental challenges, which derive from the various parameters that comprise this system, namely its configuration, the target molecule, and, of course the photoelectrode.^26^

In this regard, special efforts have been dedicated to the design and development of novel photoelectrodes based on stable and durable materials, but their cost-benefit balance is still a challenge for researchers.^27^^,^^28^ Boron-doped diamond (BDD)-based photoanodes are a prime case in point. This material is a p-type semiconductor substrate-electrode, with a reported band gap of approximately 5.5 eV.^29^^,^^30^ When its surface is physically or chemically modified with light-active semiconductors (photocatalyst), it becomes a photoelectrode, which will serve as a photoanode or photocathode according to its nature.^31^ Accordingly, modifications using BDD with BiVO_4_^32^ (n-type), ZnO (n-type),^33^ and TiO_2_ (n-type)^34^ have been used to degrade organic pollutants, although in some cases, their optical efficiency has been undercut by their relatively wide band gap, for instance, approximately 3.20 eV for the anatase phase of TiO_2_ (the most widely used photocatalyst); in other words, their optical activity is retained up to the UV region of the spectrum.^21^^,^^35^^,^^36^

In this study, synthetic TiO_2_ nanoparticles were deposited on a BDD substrate electrode using an electrophoretic deposition method.^37^ The modified heterojunction photoanode was then systematically characterized using structural, electrochemical, and photoelectrochemical techniques and its application in the PEC degradation of high-density PE (HDPE) MPs in water was examined for the first time.^27^

### BDD/TiO_2_ photoanode

#### XRD and EDS analysis

**Figure 1:**
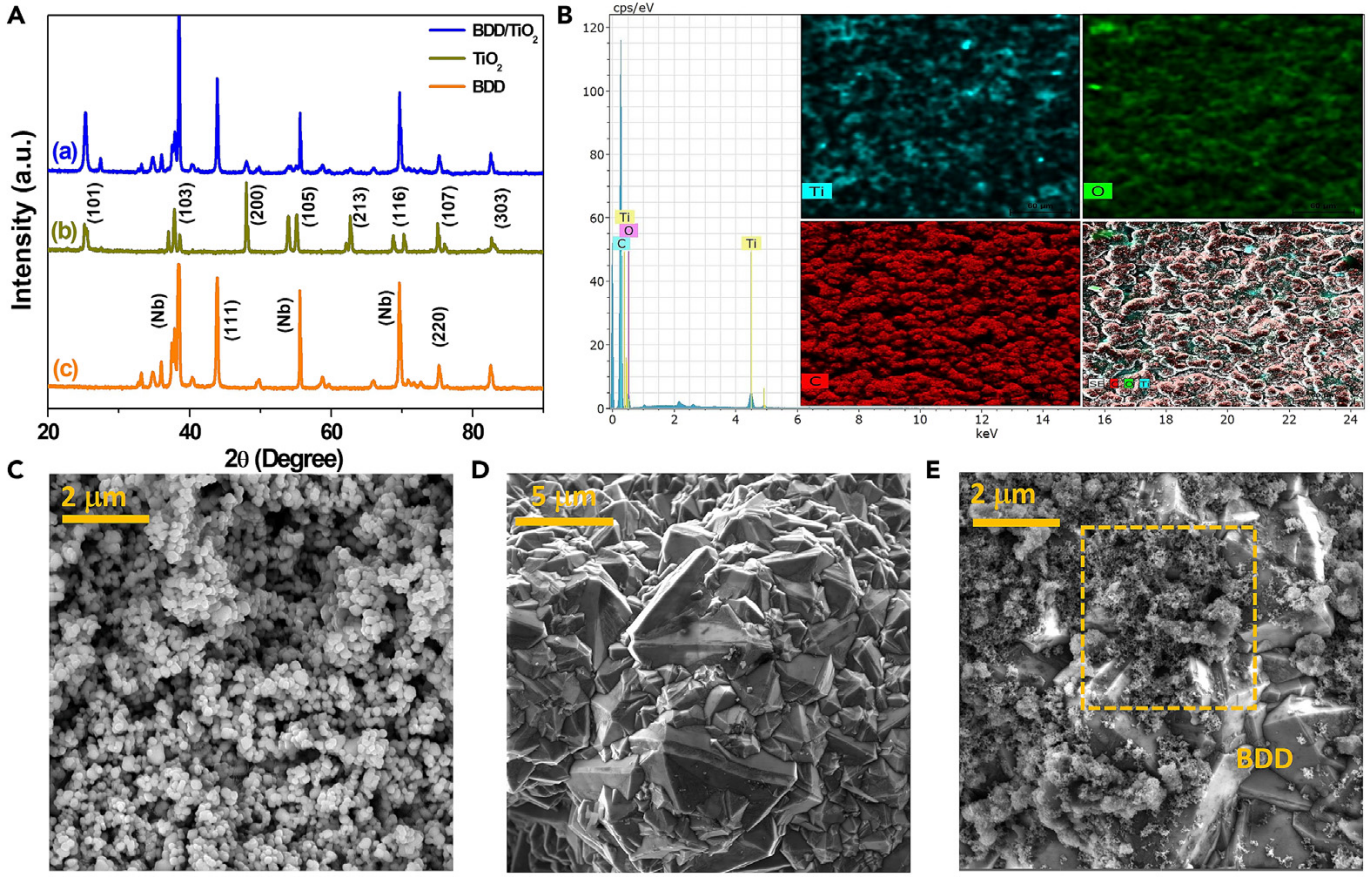
Characterization of BDD/TiO_2_ structure and morphology (A) X-Ray diffraction pattern of (a) BDD/TiO_2_ photoanode, (b) TiO_2_ anatase phase and (c) bare BDD. (B) EDS spectrum and elemental mapping of BDD/TiO_2_ photoanode. (C–E) Scanning electron microscopy images of (C) TiO_2_ anatase phase, (D) bare BDD electrode, and (E) BDD/TiO_2_ photoanode.

In order to confirm that the BDD/TiO_2_ photoanode significantly impacted the PEC and catalytic capabilities of the material and that the anatase phase of TiO_2_ had higher photocatalytic activity than the rutile phase, the surface features of the photoelectrode were evaluated.^29^^,^^38^ The X-ray diffraction (XRD) patterns of TiO_2_ Degussa P25 and the BDD/TiO_2_ photoanode produced by electrophoretic deposition is presented in Figure 1A. The various peaks in the XRD pattern in part (b) correspond to the anatase phase and the tetragonal crystal structure according to ICDD no. 900–9086. On the other hand, the peaks at 25.3°, 36.9°, 37.8°, 48.0°, 53.9°, 62.1°, 68.8°, 74.1°, and 82.2° were indexed as crystal planes (101), (103), (200), (105), (213), (116), (107), and (303), respectively.^32^ In part (c), the two distinct peaks at 43.9° and 75.3° correspond to crystal planes (111) and (220) of the diamond (ICDD no. 01-089-3441)^39^; the remaining peaks correspond to the niobium (Nb) substrate where the diamond layer was deposited. The XRD pattern of the BDD/TiO_2_ photoanode in part (a) shows that the BDD diffraction peaks at 38°, 54°, and 69° are sharp, indicating an overlap or interplay between the TiO_2_ and BDD crystal planes is possible.^40^ However, the peaks are consistent with the diffraction patterns, confirming the predominant presence of anatase crystalline phases on BDD. As shown in Figure 1, energy-dispersive X-ray spectroscopy (EDS) analysis confirmed the presence of adequately deposited TiO_2_, as well as the elemental composition. The EDS spectrum revealed the presence of C, O, and Ti in the photoanode, suggesting that the material is fairly pure, since no unwanted elements were observed. The average weight percentages were 83 %C, 15 %O, and 2 %Ti, which is consistent with the results obtained in another study.^41^ This was confirmed by scanning electron microscopy (SEM) images, presented in Figures 1C–1E. The micrograph in Figure 1C reveals the size and shape of the TiO_2_ nanoparticles; it shows a rather polydisperse grain size distribution, with particle sizes ranging from 97 to 550 nm (Figure S1A) and an irregular spherical shape. The SEM image in 1D shows the bare BDD electrode composed of randomly disordered, angular polycrystalline grains, with grain size ranging from 1.4 to 13.4 μm (Figure S1B) and uniform distribution. Finally, in Figure 1E, the BDD/TiO_2_ photoanode shows the heterojunction is well dispersed in interstitial spaces, while the photocatalyst is monodispersed.

### PEC and optical analyses

#### Electrochemical and photoelectrochemical characterization

**Figure 2:**
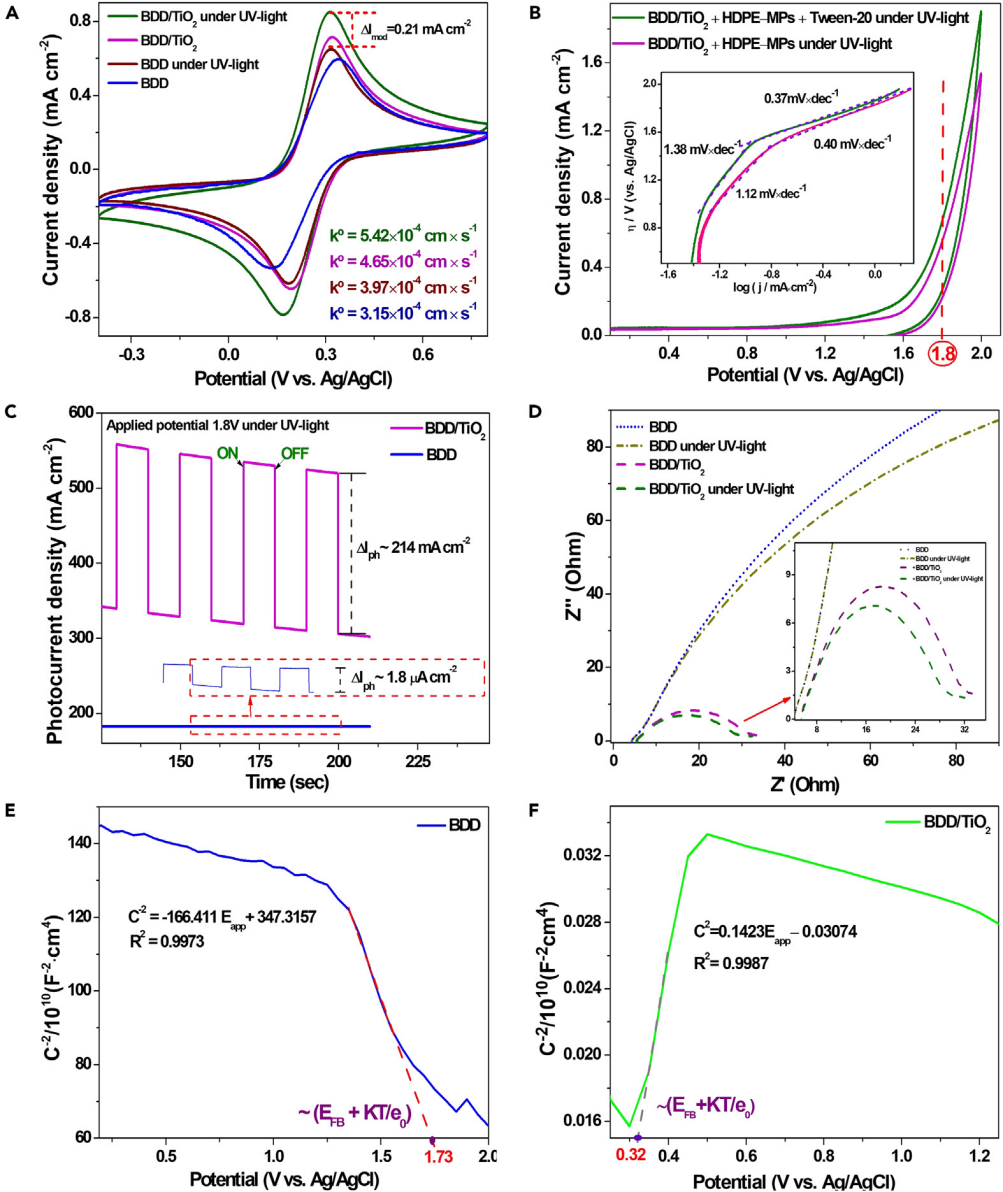
Electrochemical and photoelectrochemical characterization of BDD/TiO_2_ (A and B) Cyclic voltammetry (CV) in (A) 4.0 mmol·L-1 [Fe(CN)_6_]^3-/4-^ in 1.0 mol·L^-1^ KCl, (B) 0.1 M mol·L^-1^ Na_2_SO_4_, (inset of Tafel plot). (C) Photocurrent response in 0.1 M mol·L^-1^ Na_2_SO_4._ (D) Nyquist plot of bare BDD and BDD/TiO_2_ photoanode. (E and F) Mott-Schottky plot for (E) bare BDD and (F) BDD/TiO_2_ photoanode.

The electro- and photoelectrochemical characterization of the bare BDD electrode and BDD/TiO_2_ photoanode was done with cyclic voltammetry (CV) using the redox couple [Fe(CN)_6_]^3-/4-^. Information on the charge transfer kinetics of the bare and TiO_2_-modified BDD electrodes under both dark conditions and UV light exposure was obtained.^42^ In Figure 2A, the CV profile of the BDD electrode under UV light exhibits a slight increase in current compared to BDD under no light; moreover, the slope of the charge transfer region of the oxidation-reduction curves also increased due to the light’s effect. This further demonstrates that due to the potential region where the ferri/ferro-cyanide redox reaction occurs and the role of high-energy light such as UV, bare BDD exhibits photocurrent without needing a photocatalyst to modify its surface. Indeed, this is owed to the high content of boron impurities in the crystal lattice and the C-sp^3^/C-sp^2^ hybridization ratio on BDD.^43^^,^^44^^,^^45^ Similarly, the BDD/TiO_2_ heterojunction exhibits well-defined redox peaks in both UV light and dark conditions. The anodic peak current intensity was 0.15 mA cm^−2^ for the modified BDD electrode, but in UV light, it increased to 0.21 mA cm^−2^. Furthermore, in UV light, the anodic peak potential of BDD/TiO_2_ shifted to 0.30 V (vs. Ag/AgCl), which was 0.35 V (vs. Ag/AgCl) for the bare BDD electrode; the heterogeneous rate constant (k°) for the BDD/TiO_2_ photoanode was 5.42 × 10^−4^ cm s^−1^, which was approximately 1.7 times faster than that of the bare BDD electrode (3.97 × 10^−4^ cm s^−1^) (See also Table S1). This may be attributed to the TiO_2_ nanoparticles having a high surface area, which favors the adsorption of reagents on the photoelectrode surface, which enhances the charge transfer process.^41^^,^^46^^,^^47^

Additionally, the CV profile in Figure 2B demonstrates HDPE-MPs’ influence on the photoelectrochemical response of the BDD/TiO_2_ photoanode in the electrolyte medium. However, there is no extra oxidation peak, suggesting that MPs do not undergo direct oxidation; therefore, their oxidation is expected to occur mainly through PEC. Conversely, the HDPE MPs’ high hydrophobicity requires a surfactant to decrease the surface tension between the aqueous medium and polymer to achieve a proper dispersion. Accordingly, the controlled addition of tween 20 (8 μmol L^−1^) is needed, which causes a subtle increase in the medium’s resistance (a slight decrease in conductivity).^48^^,^^49^ However, because Tween 20 is also sensitive to oxidation at the electrode surface, a faradaic current contribution corresponding to this reaction occurs, which is indicated by a slight current increase in the voltammetric profile, shown in Figure 2B.^50^^,^^51^ Furthermore, the behavior of the BDD/TiO_2_ under UV light improved the current response of the oxidation peak, because the onset potential (V_onset_) for the HDPE MPs and Tween 20 mixture (∼ 1.65 V (vs. Ag/AgCl)) was lower than that for HDPE MPs alone (∼ 1.73 V (vs. Ag/AgCl)).

The inset of Figure 2B shows the Tafel plot analysis from the voltamperometric data; two slopes are visible, attributed to independent charge transfer processes. At an overpotential greater than 1.8 V (vs. Ag/AgCl), the oxygen evolution reaction (OER) is expected to be dominant; accordingly, prior to the OER, ^•^OH generation was assumed to dominate, which was further verified via radical trapping analysis (see the “^•^OH trapping study” section). The Tafel analysis is critical as it allows the proper selection of the anodic overpotential value with the highest expected catalysis yield of ^•^OH radicals and thus an effective degradation of organic matter.^23^ However, because of changes in cell configuration, such as increased volume or distance between electrodes in the PEC treatment, it is not uncommon to use overpotentials even higher than those predicted by Tafel analysis. Na_2_SO_4_ (concentration 0.1 mol L^−1^) was ultimately selected for the electrochemical tests given its stability and its use in other MPs degradations.^49^^,^^52^^,^^53^

In this regard, the transient photocurrent response at a bias potential of +1.8 V (vs. Ag/AgCl) was evaluated according to the CV profile under UV light (Figure 2C). The highest photocurrent density was 214 mA cm^−2^, which corresponded to the BDD/TiO_2_ photoanode; this was significantly higher than that of the bare BDD (1.8 μA cm^−2^), indicating that the construction of the p-n heterojunction of BDD/TiO_2_ favored the charge transfer across the interfaces of the two semiconductors and greatly inhibited the rapid recombination of photogenerated $e_{\text{CB}}^{-}/h_{\text{VB}}^{+}$ pairs.^54^

To determine the drop in charge transfer resistance (R_ct_) from electrode modification, Nyquist analysis was used (Figure 2D).^55^ Hence, in the Nyquist plot, the radius of the semicircle indicated a significant decrease in the photoanode’s R_ct_ (23 Ohms) compared to the bare electrode, and a decrease still occurred under UV light conditions; in other words, the presence of UV light increased the photoanode’s internal electron mobility, which enhanced the charge dynamics and led to a decrease in the instantaneous recombination rate of $e_{\text{CB}}^{–}/h_{\text{VB}}^{+}$ pairs. To assess the interfacial charge transfer and recombination, Mott-Schottky plots for BDD and TiO_2_ were calculated. Figure 2E indicates the respective p-type and n-type semiconducting natures of BDD and TiO_2_. The capacitance-potential curve also demonstrates that a p-n heterojunction successfully formed between TiO_2_ and BDD; conversely, the flat band potential (E_fb_) was calculated from the extrapolation of the linear region of the x axis, and donor or acceptor density (N_D_ or N_A_) was calculated from the slope with Equation 1:^56^

(Equation 1) $$\frac{1}{C_{\text{SC}}^{2}}=\pm \frac{2}{A{\varepsilon \varepsilon }_{0}\text{eN}}\left(E_{\text{app}}–{\;E}_{\text{FB}}\;–\;\frac{k_{B}T}{e}\right)$$

where C_sc_ is the space charge capacitance; *e* is the elementary charge; *N* is the carrier concentration for the acceptor or donor (cm^−3^); E_app_ is the applied bias potential; ***ε*** is the dielectric constant of the material and has a value of 55 and 5.6 for TiO_2_ and BDD, respectively; ***ε***_0_ is the vacuum dielectric constant (8.85 × 10^−14^ F cm^−1^); k_B_ is the Boltzmann constant (1.38 × 10^−23^ J K^−1^); *T* is the absolute temperature; and *A* is a material-specific constant.^29^^,^^56^

The E_fb_ of TiO_2_ was 0.32 V (vs. Ag/AgCl) with a positive slope (n-type semiconductor), and the E_fb_ of the BDD was 1.73 V (vs. Ag/AgCl) with a negative slope (p-type semiconductor), which are consistent with previously reported values.^29^^,^^56^^,^^57^ This observation was further validated by the carrier density of BDD/TiO_2_ (4.6 × 10^15^ cm^−3^). Further, the anticipated upward shift of the Fermi level caused by the elevated charge carrier density may lead to a noticeably greater degree of band bending at the heterostructure’s surface, which would encourage charge separation at the interface between the heterostructure and electrolyte.^57^

#### Optical properties

**Figure 3:**
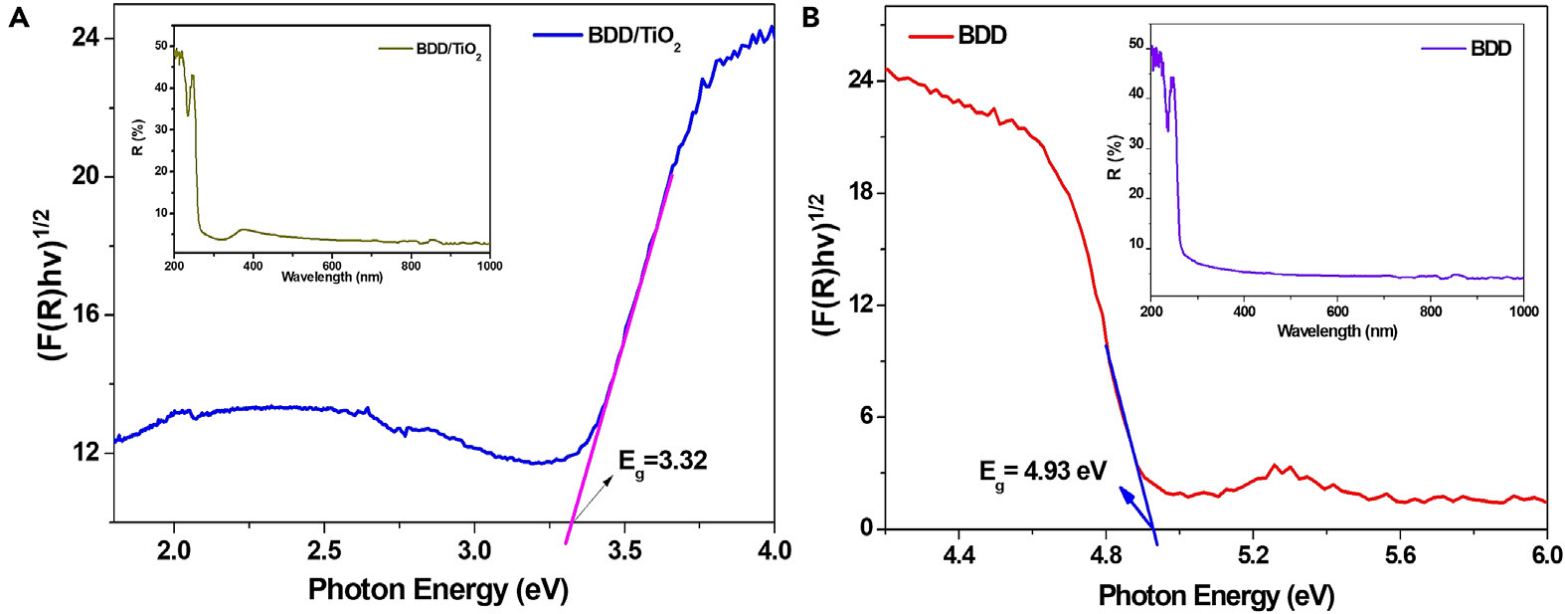
Optical analysis of BDD/TiO_2_ photoanode (A and B) Band gap and UV-vis diffuse reflectance spectra (insets) of (A) BDD/TiO_2_ and (B) bare BDD.

Figure 3 shows the diffuse reflectance spectra (inset) and Tauc plot for determining the band gap energy of the bare BDD and BDD/TiO_2_ photoanode according to Equation 2:^32^

(Equation 2) $$\left(F\left(R\right)hv\right)=A{\left(hv\;–\;E_{g}\right)}^{n/2}$$

Where F(R) is the Kubelka-Munk function, hv is the photon energy, *A* is a constant, *n* is an integer that depends on the transition (1 for direct and 4 for indirect), and E_g_ is the band gap of the semiconductor. TiO_2_ and BDD are indirect transition semiconductors; therefore, (F(R) hv)^1/2^ vs. hv was plotted, from which the values of E_g_ for the TiO_2_ and BDD electrodes were estimated as 3.32 eV and 4.93 eV, respectively. The band gap energy values for the heterojunction are consistent with previously reported values for the semiconductors.^46^^,^^58^^,^^59^^,^^60^

### Photoelectrocatalytic degradation of HDPE-MPs

#### FTIR analysis

**Figure 4:**
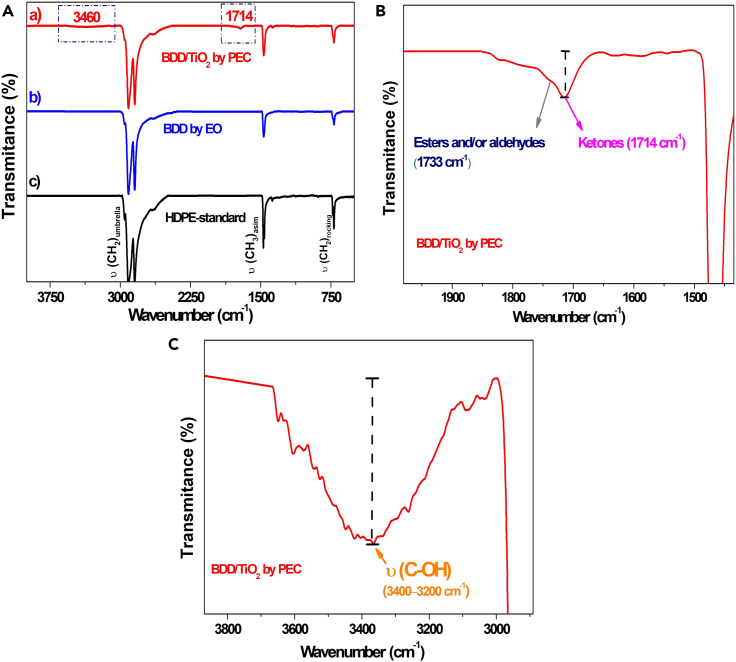
Performance degradation of high-density polyethylene microplastics on BDD/TiO_2_ (A) FTIR spectra of HDPE-MPs before and after 10 h reaction by EO using bare BDD and PEC using BDD/TiO_2_ photoanode. (B and C) Magnified spectra of HDPE-MPs after PEC treatment.

Fourier transform infrared spectroscopy (FTIR) was used to quantify the carbonyl content of HDPE MPs in a spectral ranging range from 4000 to 650 cm^−1^. The signal was averaged over 10 scans at a resolution of 4 cm^−1^. As shown in Figure 4A, the characteristic peaks of –CH_2_ in the HDPE MPs are not missed after EO and PEC treatments. The original polymer (spectrum c, Figure 4A) shows typical peaks at 2850 cm^−1^ and 2912 cm^−1^ corresponding to C–H stretching in –CH_2_ groups. In addition, at 1470 cm^−1^ there is a bending band of CH_2_ groups.^61^^,^^62^

Additionally, to monitor the oxidation and deterioration of the mechanical properties, we concentrated on the stretching vibrations of the carbonyl and hydroxyl groups. The carbonyl index (CI) is used to determine the degree of oxidation or degradation of certain materials, such as PE MPs, based on the absorbance ratio between the carbonyl group at 1714 cm^−1^ to that of a reference band (1500 and 1400 cm^−1^).^63^ Comparing the spectra before and after electrochemical oxidation, no peak at 1714 cm^−1^ was observed that corresponded to the (–C = O) bond, and it is possible the increase of the hydroxyl region or hydroxyl index (HI) that appeared at 3460 cm^−1^ was due to a typical intermolecularly bonded –OH and free OH, which has been found by previous studies.^49^^,^^52^

Regarding the PEC treatment, as shown in Figure 4A part (a) and in the magnified spectrum in Figure 4B, a significant increase in the CI was determined, from 0.006 to 0.08, indicating the generation of excited states in the HDPE MPs, leading to the cleavage of the polymer chains and the formation of (–C = O), (–C–O), and (–COOH) bonds.^63^ Moreover, an increase was highlighted, from 0.48 to 0.61, which facilitated the formation of new groups (–OH and/or –OOH), which were subject to further conversion of carbonyl groups (Figure 4C).^64^ This was confirmed by gravimetric (Figure S3) and thermogravimetric (Figures S4A and S4B) analysis when applying a current density of 6.89 mA cm^−2^. The percentage degradation of MPs-PE was 58.0% ± 0.1 and 89.9% ± 0.1, by electrooxidation and PEC, respectively (Table 1). In contrast, thermogravimetric analysis of Figure S4A shows the change in polymer mass before and after treatment by PEC.^65^ The decomposition weight loss of HDPE-MPs occurred in a first stage at approximately 424°C, and had the highest weight loss at approximately 466°C with complete decomposition of the polymer microspheres,^66^ (See also Tables S2 and S3; Figure S4A). In summary, the weight loss after HDPE MP degradation via PEC occurred at a lower temperature than for standard MPs owing to the presence of ketones/carboxylic acids in polymer chains, which are first to leave in thermal decomposition.

**Table 1:** Percentage weight loss, TOC and COD analysis using EO and PEC

Current density (mA·cm^-^^2^)	EO			PEC		
	Degradation (%)	TOC (mg L^−1^ O_2_)	COD (mg L^−1^ O_2_)	Degradation (%)	TOC (mg L^−1^ O_2_)	COD (mg L^−1^ O_2_)
1.49	17.58 ± 0.03	3.6	9	23.58 ± 0.05	10.5	26.5
6.89	57.97 ± 0.09	250	633	89.91 ± 0.08	396.2	1003
9.52	55.48 ± 0.12	11.1	28.2	83.98 ± 0.07	1.7	4.2

TOC, total organic carbon; COD, chemical oxygen demand; EO, electrochemical oxidation; PEC, photoelectrocatalysis.

### *SEM*

**Figure 5:**
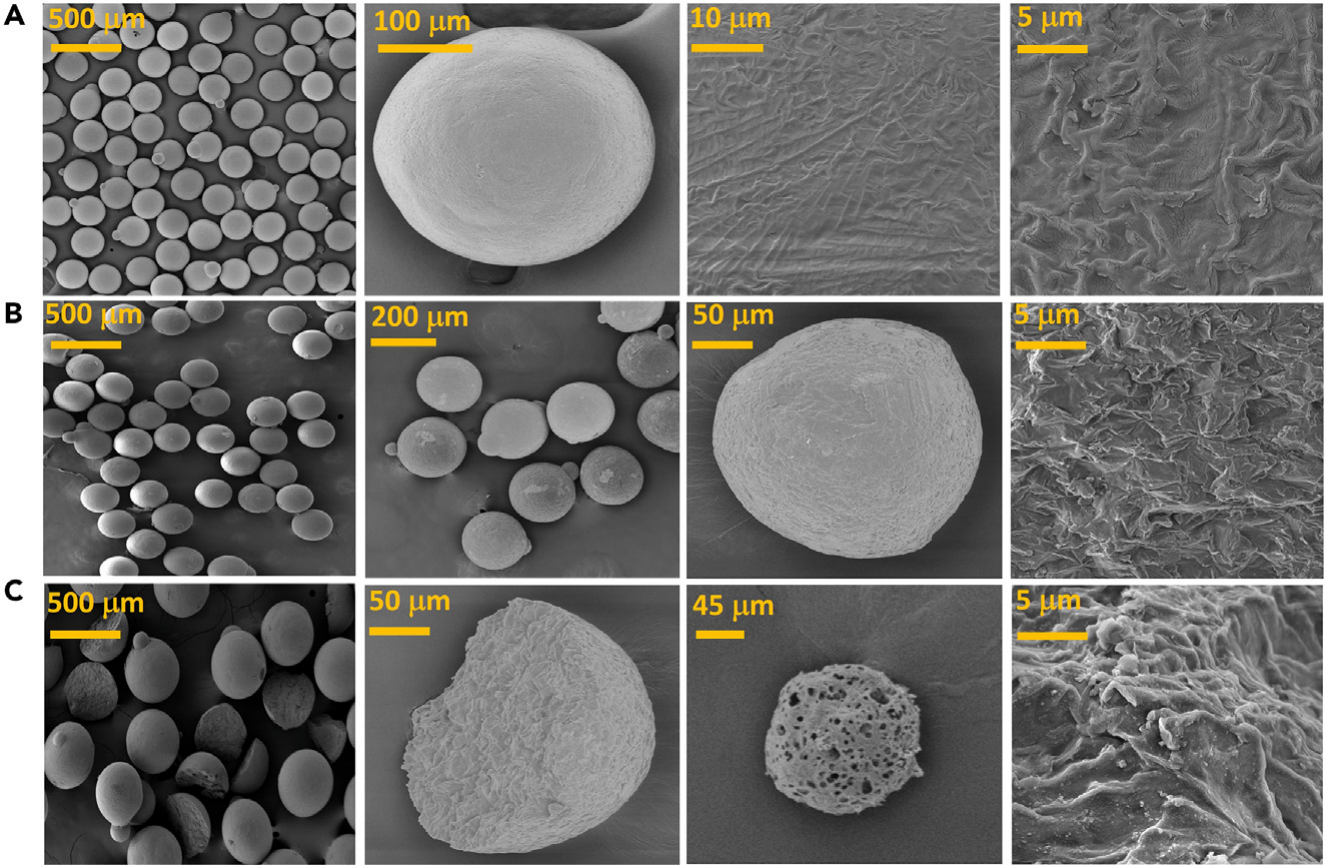
SEM images of high-density polyethylene microplastics at different scales (A) Before treatments. (B) After electrochemical oxidation (10 h reaction). (C) After photoelectrocatalysis (10 h reaction).

Based on the aforementioned tests, SEM analysis was performed to determine the change in MP morphology during the electrochemical oxidation and PEC processes. As seen in Figure 5A, the standard MP was a compact sphere with a smooth surface with little roughness and an approximate diameter of 250 μm. After electrochemical oxidation (Figure 5B), the MPs showed an amorphous spherical shape; the presence of surface cracks and cavities indicated that the degree of deterioration increased, as ^•^OH could enter the surface of the HDPE-MPs at a greater depth.^67^ During PEC, visible changes in the surface microstructure occurred (Figure 5), which decomposed into hemispheres and fragments, possibly due to the interaction (collisions) between the BDD/TiO_2_ photoanode and HDPE-MPs under UV light. Furthermore, a honeycomb-like porosity with a network of interconnected voids was visible (Figure 5C, scale 45 μm), indicating PEC achieved maximum degradation of the HDPE-MPs.

### *TOC and COD analysis*

Total organic carbon (TOC) and chemical oxygen demand (COD) analyses were carried out on the remaining solution that comes from degradation process once the HDPE-MPs were filtered. Thus, TOC analysis insights into the breakdown and the transformation of organic compounds associated with microplastic particles after the degradation process.^68^ In the same way, COD analysis measures the amount of oxygen required to oxidize the organic matter released by microplastics degradation and can be used to evaluate the organic pollution load.^69^ These analyses can provide useful information regarding degradation methods effectiveness and the extent to which MPs are breaking down into smaller compounds.

**Figure 6:**
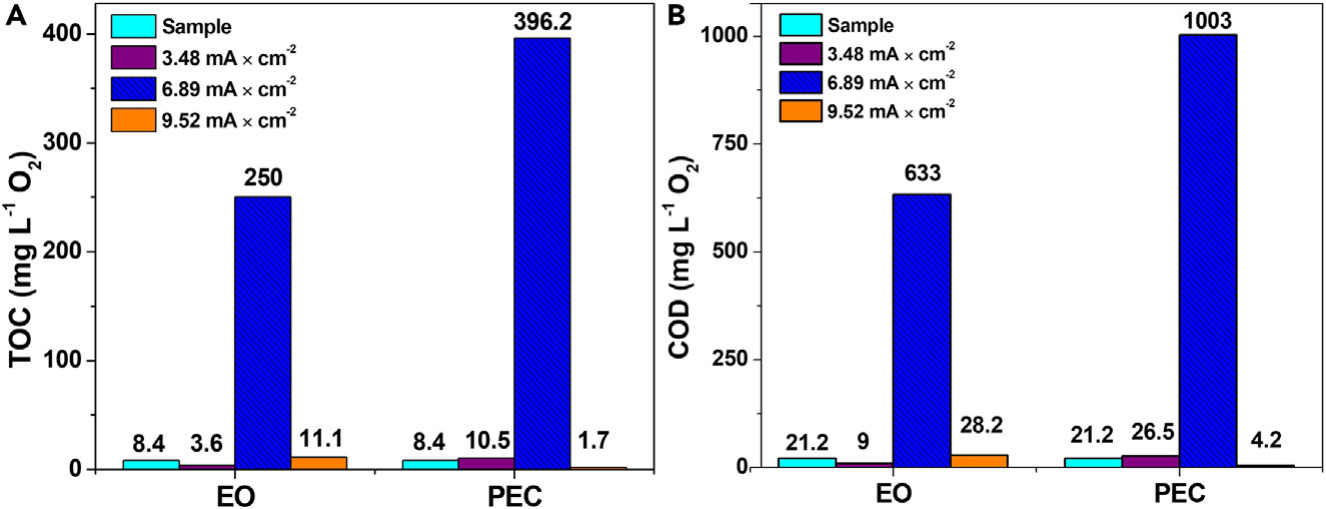
Analysis of the filtered solution of the samples treated at different current densities (A) Total organic carbon obtained (TOC). (B) Chemical oxygen demand (COD).

Therefore, TOC and COD were carried out using three different current densities (Figure 6); the highest amount of organic matter from polymer chain cleavage corresponded to 6.89 mA cm^−2^ with 250 and 396.2 mg L^−2^ O_2_ for EO and PEC, respectively. Using the same current values for COD, 633 and 1003 mg L^−2^ O_2_ were obtained for EO and PEC, respectively, which were the largest amounts of oxygen from the treatment (See also Table S4). Namely, the formation of oxidized products, such as aldehydes and ketones in steps 4.1 and 4.2 (suggests that HDPE-MPs chains are breaking down. This highlights a transformative process in which the HDPE molecules suffer chemical changes leading to the generation of oxygen-containing functional groups.

**Figure 8:**
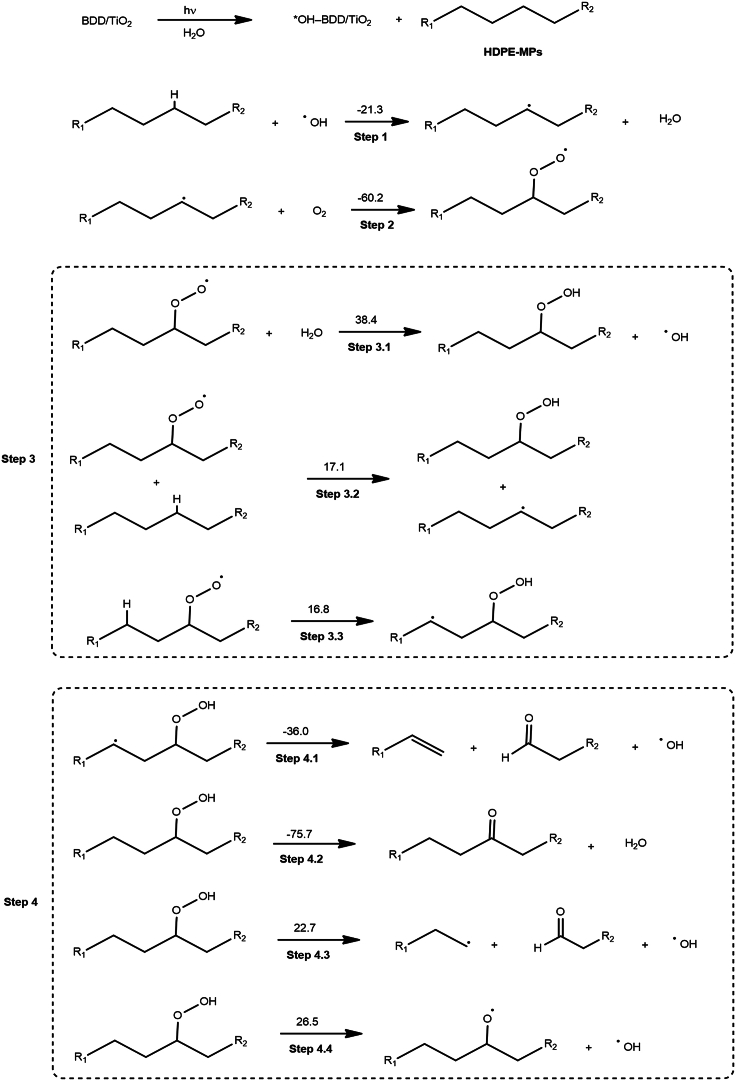
Proposed mechanism of degradation of high-density polyethylene microplastics by photoelectrocatalysis Values above the arrows are the free energy change of each case in kcal·mol^-1^.

### ^•^OH trapping study

**Figure 7:**
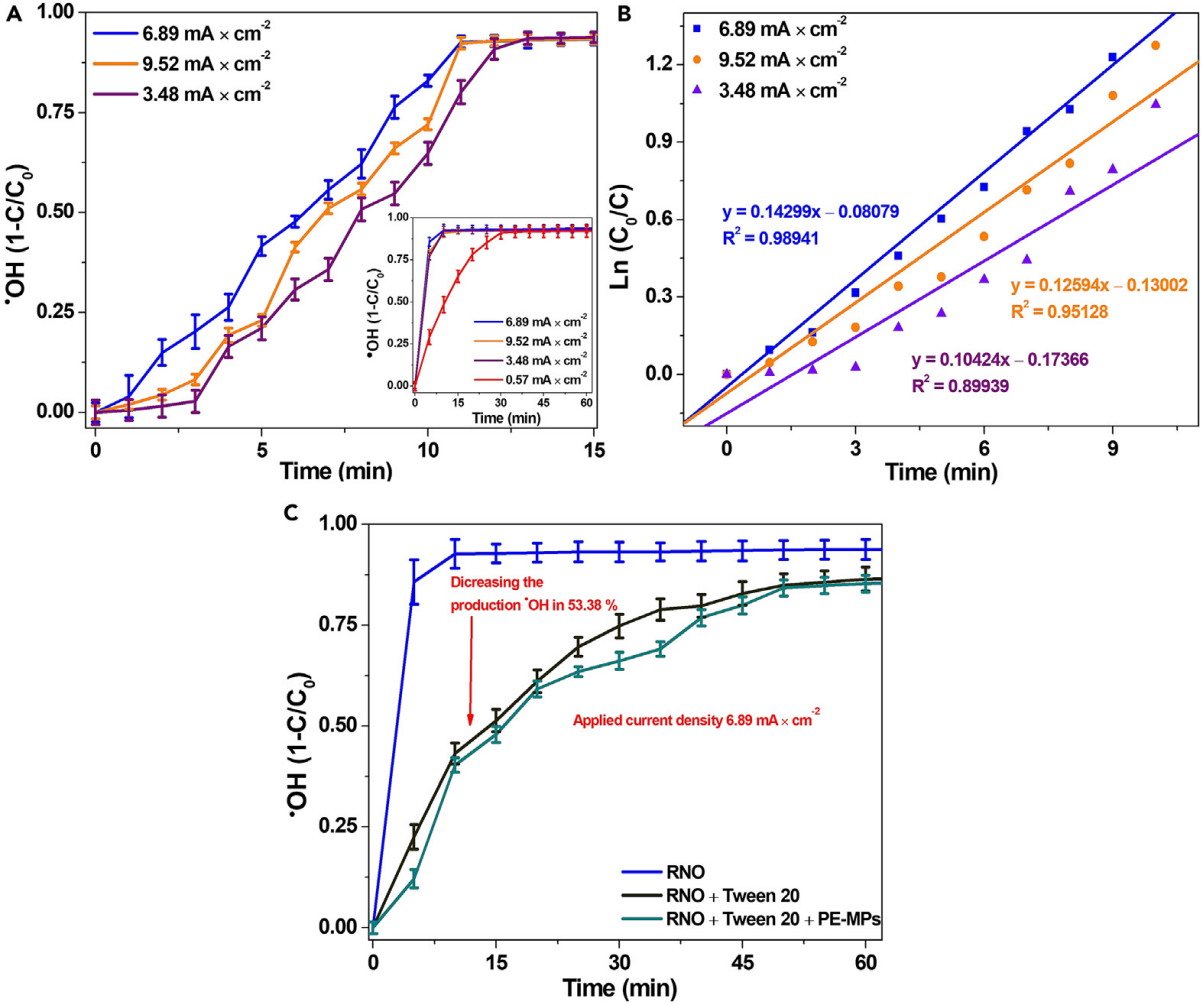
Analysis of ^•^OH generation in the absence and presence of HDPE MPs (A) ^•^OH at different current densities in absence of high-density polyethylene microplastics (HDPE MPs). (B) First-order reaction kinetics analysis of RNO (0.1 mol⋅L^-1^ Na_2_SO_4_). (C) Effect of the presence of HDPE MPs and Tween 20 on the production of ^•^OH. Data are represented as mean ± SEM.

Since photoelectrodegradation of pollutants is usually mediated by ROS, mainly ^•^OH is important for all analysis.^70^ In this regard, the quantification of hydroxyl radicals though spectroscopy was used to determine the maximum level of HDPE-MPs degradation by this highly oxidizing reactive species. Figure 7A shows the ^•^OH generated in the absence of HDPE-MPs at the current densities of 3.48, 6.89, and 9.52 mA cm^−2^ during 15 min of PEC; the inset presents the same current densities as well as that of 0.57 mA cm^−2^, which corresponds to a 1.8 V vs. Ag/AgCl and 60 min duration and was the reference initial potential of ^•^OH production. These values were consistent with the Tafel analysis. The *N, N*-dimethyl-4-nitroso-aniline (RNO) disappearance rate follows first-order kinetics that is the maximum amount of ^•^OH occurs at 6.89 mA cm^−2^, with a kinetic constant of 0.143 min^−1^. At 9.52 mA cm^−2^, the reaction rate slows, and O_2_ evolution is predominant, which does not favor the degradation process and implies a higher energy consumption (Figure 7B).^71^^,^^72^

The amount of ^•^OH photoelectrogenerated in the presence and absence of HDPE-MPs was compared at a current density of 6.89 mA cm^−2^. According to Figure 7C, with MPs, the ^•^OH concentration decreased by 53% in 100 min compared to when MPs were absent, that is, a fraction of the generated ^•^OH was used for MPs degradation. Conversely, when Tween 20 was evaluated with RNO, the ^•^OH production deceased because a fraction was used for surfactant degradation, and it is possible that solution resistance also increased.

### Proposed mechanism

Based on this study’s analyses, we propose a possible degradation pathway of HDPE MPs (Figure 8). As the first step, the C–H bond of the HDPE molecule is broken by the ^•^OH generated by PEC on the BDD/TiO_2_ surface, causing the production of a free radical centered on the carbon atom of the HDPE molecule.^73^ Thereafter, each reaction step was computationally evaluated and the free energy change for each was estimated. Steps 1 and 2 involve the abstraction of the H from the polymer and the incorporation of the O_2_ molecule, resulting in free energy changes of −21.3 and −60.2 kcal mol^−1^, respectively. These negative values indicate highly favorable and exergonic reactions.^74^ In step 3, three reaction paths for the formation of the hydroperoxide species are suggested and analyzed. Computational results reveal that step 3.1 is not plausible, considering a free energy change of 38.4 kcal mol^−1^.^75^ However, for steps 3.2 and 3.3, comparable values for the free energy change (approximately 17 kcal mol^−1^) were obtained, suggesting that both processes are favorable and may occur as parallel reactions. A free energy change of about 17 kcal mol^−1^ is reasonable for an oxidation process performed at room temperature. In step 4, four competitive reactions are proposed for the decomposition of the hydroperoxide species.^76^ Examining the free energy change values, it becomes evident that steps 4.1 and 4.2 represent the most feasible pathways, with values of −36.0 and −75.7 kcal mol^−1^, respectively. These results indicate that the direct formation of aldehydes and ketones from the hydroperoxide species is more likely, and no homolytic dissociation of the hydroperoxide is necessary (as suggested in step 4.4).^77^ In detail, step 4.1 involves a beta cleavage leading to the formation of an aldehyde or ketone, with a subsequent release of a hydroxyl radical. Similarly, a beta-cleavage aided by another radical in the system is suggested for step 4.2. Table S5 lists the Cartesian coordinate data used in the modeling.

### Conclusion

PEC using a BDD/TiO_2_ photoanode is an efficient technology for the degradation of HDPE-MPs in aqueous media. Photoelectrocatalytic performance in an electrolyte medium of 0.1 mol L^−1^ Na_2_SO_4_ and an optimum current density setting of 6.89 mA cm^−2^ during 10 h reaction achieved a high degradation efficiently (89.91 ± 0.08). FTIR results showed that a partial oxidation of the microplastics leads to the formation of organic species with carbonyl groups, while SEM results show the polymer with an amorphous spherical shape with surface cracks and cavities. Furthermore, evaluating the production of ^•^OH radicals at the optimum current density showed that the oxidation of the microplastics was mediated by this oxidizing species, which was supported by density functional theory (DFT) calculations of the proposed degradation mechanism. Ultimately, the photoelectrocatalytic efficiency with BBD/TiO_2_ for the degradation of HDPE-MPs proved to be more efficient than electrochemical oxidation.

### Limitations of the study

In this work we have evaluated a BDD/TiO_2_ photoanode in the photoelectrocatalytic degradation of HDPE-MPs in a laboratory scale system. We have found the optimal parameters to obtain the highest efficiency of the process, and we have used computational computations based on DFT to assess the degradation mechanisms. In this sense, we consider it necessary for future research to prove different visible light active photoanodes, and to extend their application to other microplastics. In addition, it is recommended to integrate analytical techniques for the identification and quantification of by-products to strengthen the theoretical investigation of the degradation mechanism. In addition, it is recommended to study possible value-added by-products. This research is a first approximation at the laboratory level of the use of PEC in the degradation of microplastics. It is a field with a lot of potential that requires further in-depth studies for its application on a large scale.

## STAR★Methods

### Key resources table

**Table:** 

REAGENT or RESOURCE	SOURCE	IDENTIFIER
**Chemicals, peptides, and recombinant proteins**		

Sodium sulfate	Sigma Aldrich	CAS 7757-82-6
Sulfuric acid	Sigma Aldrich	CAS 7664-93-9
Potassium ferricyanide	Sigma Aldrich	CAS 13746-66-2
Potassium ferrocyanide	Sigma Aldrich	CAS 14459-95-1
*N,N*-Dimethyl-4-nitroso-aniline	Merck	CAS 138-89-6
Degussa P25 titanium dioxide nano powder	Sigma Aldrich	CAS 13463-67-7
Tween 20 biocompatible surfactant	Cospheric	CAS 9005-64-5
High density polyethylene microspheres (0.96 g/cc 212–250 μm)	Cospheric	CAS 9002-88-4
BDD/Nb	Metakem™	Germany
Cellulose filter	CHMLAB GROUP	pore size 0.22 μm

**Software and algorithms**		

Origin Pro 8.9	OriginLab Corporation	Massachusetts, USA
Gaussian 16	Materials Studio	Gaussian®

**Other**		

AC/DC Power supply	BK Precision	1760A
Scanning electron microscopy	TESCAN	TESCAN MIRA 3
X-ray diffraction	PanAnalytical	PanAnalytical Empyrean
Electrochemical workstation	CH Instruments	CH 1230
Germicidal LED lamp	Sylvania germicidal	21 W UV-C
Diffuse reflectance spectroscopy	Horiba	Horiba Micro HR monochromator coupled DSS-SIGA (2.2)020A
Fourier transfer infrared spectroscopy	JASCO	JASCO FT/IR-6800 Type A spectrophotometer
Thermogravimetric analysis	TGA-METTLER TOLEDO	IST16-GC/MS 1
Total Organic Carbon analysis	Shimadzu TOC-5050	Method-5310B
Chemical Oxygen Demand analysis	HACH 8000	Usepa HACH-2000
UV-vis spectroscopy	Agilent technologies	Cary 60
UV LED lamp	Cole-Palmer	T8 20W 110V 245 nm

### Resource availability

#### Lead contact

Further information and requests for resources should be directed to and will be fulfilled by the lead contact, Patricio J. Espinoza-Montero (pespinoza646@puce.edu.ec).

#### Materials availability

This study did not generate new materials.

#### Data and code availability

• This article includes all datasets generated or analyzed during this study.
• Any additional information required to reanalyze the data reported in this paper is available from the lead contact upon request.
• This paper does not report original code.

### Method details

#### Preparation of BDD/TiO_2_ photoanode

The BDD electrode (Metakem, Germany; 40 mm × 15 mm double-sided) was electrochemically activated prior to use by anodic polarization in 0.5 M H_2_SO_4_ (Sigma-Aldrich 98%) at 50 mA in a BK Precision AC/DC power supply during 30 min, the electrode was rinsed with plenty of water an air dried. The BDD/TiO_2_ photoanode was prepared by electrophoretic deposition reported in our previous work with slight modifications.^37^ Briefly, a 2.5 w/v% suspension of Degussa P25 TiO_2_ nanopowder (Sigma-Aldrich, particle diameter <25 nm) was prepared in a 2.5 v/v% isopropanol-water mixture. Electrophoretic deposition was carried out in a two-electrode system using an anode of aluminum plate and the BDD electrode as the cathode at a 10 mm spacing. Both electrodes were immersed in the as-prepared TiO_2_ suspension under magnetic stirring and a 4.5 V bias was applied during 15 s. Finally, the TiO_2_-modified BDD electrode was air dried at 100°C on a heating plate for 5 min, and then calcined in an oven at 200°C for 12 min in order to improve the adhesion to the BDD substrate.

#### BDD/TiO_2_ photoanode characterization

The morphology and elemental mapping of the as-prepared photoanode was characterized by scanning electron microscopy coupled to electron dispersive spectroscopy (EDS) (SEM, TESCAN MIRA 3). The structure and crystal phases were identified by X-ray diffraction using a PanAnalytical Empyrean X-ray diffractometer.

For the photo(electro)chemical experiments, a CH Instruments 1230C workstation was used, using a typical three-electrode arrangement. The BDD or BDD/TiO_2_ photoanode as the working electrode, a Pt mesh as counter electrode, and Ag/AgCl as reference electrode. A 40 mL quartz cell was used and confined in a dark box to control the assays in darkness and under illumination. Cyclic voltammograms were recorded in 2.0 mM ferri/ferrocyanide redox couple (K_3_[Fe(CN)_6_]/K_4_[Fe(CN)_6_], Sigma-Aldrich 98%) in 1.0 M KCl (Sigma-Aldrich 99%) at sweep rate of 100 mV s^−1^, and CV profiles were also recorded in the presence of only electrolyte (0.1 M Na_2_SO_4_, Sigma-Aldrich 99%) to determine the photocurrent potentials. For the photocurrent transients, the cell was illuminated with a 21 W UV-C germicidal LED lamp in a chopped-light (ON-OFF) system. Electrochemical impedance spectra were obtained in a frequency range from 10000 to 0.1 Hz in 0.1 M Na_2_SO_4_ at a potential of 1.8 V vs. Ag/AgCl under light and dark conditions according to the voltammetric profile. Mott-Schottky analyses were performed in the presence of ferri/ferrocyanide redox couple.

A multifunctional optical system was used to record the diffuse reflectance spectra. A Horiba Micro HR monochromator was used coupled to a Horiba DSS-SIGA (2.2)020A dual sensor (Si over InGaAs). The signals were digitalized with a data acquisition system (SpectrAcq3, Horiba) controlled by scientific software (SynerJY, Horiba). An optical fiber-guided light from a Xenon arc lamp (75W, PowerArc, PTI, Horiba) continuous source and the Si sensor were used. The spectra were recorded in the range from 200 nm to 1000 nm.

#### Degradation of HDPE-MPs

High-density polyethylene microplastics (HDPE-MPs) were purchased from Cospheric LLC, USA (density: 0.96 g cm^−3^, diameter: 212–250 μm) and were used without further cleaning or treatment. For degradation, a 0.1 w/v% suspension of HDPE-MPs was prepared in 0.1 M Na_2_SO_4_ electrolyte solution, and 8 μmol L^−1^ tween 20 (Sigma-Aldrich, biocompatible surfactant) was added to enhance the quality of suspension. The degradation reaction was carried out in a quartz cell with a volume of 80 mL during 10 h in a two-electrode system: bare BDD or BDD/TiO_2_ as the anode and a Pt mesh as the cathode. The system was illuminated with a 20 W UV lamp (254 nm) and the cell was confined in a dark box to have further control of light and dark conditions. Three different current densities (3.48, 6.89 and 9.52 mA cm^−2^) were tested using a BK Precision AC/DC power supply. The anode potential was monitored by connecting an AC/DC ZOTEK TM ZT 102 multimeter in series with the Ag/AgCl reference electrode. The temperature was maintained during all degradations at 25 ± 3°C and the system was kept under magnetic stirring at 600 rpm throughout the experiments. Degradations of HDPE-MPs were performed by electrooxidation with the bare BDD electrode and by photo(electro)oxidation with BDD/TiO_2_ photoanode.

#### Microplastics characterization

After the degradation experiments, the microplastics were collected by vacuum microfiltration using a cellulose filter (CHMLAB GROUP, pore size 0.22 μm) and dried at 100°C during 2 h. Samples from three degradation experiments were collected to ensure adequate amount of microplastics for further analysis. Morphological changes of HDPE-MPs were investigated by SEM. The samples were prepared by sputtering with gold prior to analysis. The surface functional groups of microplastics were examined by Fourier transform infrared spectroscopy using a JASCO FT/IR-6800 Type A spectrophotometer. Carbonyl index (CI) and the Hydroxyl index were calculated according by Almond^62^ and Campanale.^63^^,^^64^

$$\text{Carbonyl}\;\text{index}\;\left(\text{CI}\right)=\frac{\text{Area}\;\text{under}\;\text{band}\;1850-1650{\text{cm}}^{-1}}{\text{Area}\;\text{under}\;\text{band}\;1500-1420\;{\text{cm}}^{-1}}$$

$$\text{Hydroxyl}\;\text{index}\;\left(\text{HI}\right)=\frac{\text{Area}\;\text{under}\;\text{band}\;3353-3021{\text{cm}}^{-1}}{\text{Area}\;\text{under}\;\text{band}\;1504-1467\;{\text{cm}}^{-1}}$$

Thermogravimetric analysis was carried out on a TGA-METTLER TOLEDO in a temperature range from 25°C to 900°C, in a nitrogen atmosphere and at a heating ramp of 10°C per min. The efficiency of HDPE-MPs degradations was determined by gravimetric analysis following the method proposed by Ariza.^15^ In brief, the remaining filtered microplastics were weighed and percentage of degradation was calculated by:

$$\text{MPs}\;\text{degradation}\;\left(\%\right)=\frac{M_{0}\;–\;M_{t}}{M_{0}}\times 100$$

Where M_0_ is the initial mass of HDPE-MPs and M_t_ is the mass after 10 h reaction. Finally, the remaining solution was analyzed by measuring the total organic carbon (TOC) produced by the degradation in the non-dispersive infrared detection method-5310B in an SHIMADZU TOC-5050, and the chemical oxygen demand (COD) of the same remaining solution was tested by the HACH 8000 method in a USEPA HACH-2000 spectrophotometer.

#### Trapping of hydroxyl radicals

In order to determine the maximum hydroxyl radical production, the RNO (*N, N*-Dimethyl-4-nitroso-aniline purchased from Merck) trapping probe method was used.^72^ In essence, quantification was performed by UV-vis spectroscopy using a Cary 60-Agilent technologies Inc. spectrophotometer. Absorbances and then concentrations were measured at a maximum wavelength of 440 nm, which corresponds to absorption band of RNO-OH compound. Finally, the relative OH concentration was calculated by:

$$\cdot \text{OH}=1\;-\;\frac{C}{C_{\text{o}}}$$

Where ^·^OH is the relative concentration of hydroxyl radicals, C_0_ is the RNO initial concentration (0.03 mmol L^−1^) and C is the RNO concentration at each time point. All trapping experiments were carried out at the different current densities tested in degradation, in presence and absence of the microplastics and tween 20.

#### Theoretical calculations

The computations were executed using Gaussian 16 with Density Functional Theory (DFT). The selected theoretical level, ωb97xd/6–311 + g(d,p), was chosen due to its superior performance in capturing weak interactions considering de dispersion within the functional’s mathematical framework.^73^^,^^74^^,^^75^ This method was consistently applied to all stationary points throughout the reaction. Optimization calculations were employed to minimize the system’s energy and obtain the most stable structures for each point. The following optimization and frequency, performed with default parameters, were used to estimate absolute free energy using the statistic thermodynamic approach. These values were then used to determine free energy changes involved in each suggested step for the proposed mechanism. Solvent effects were considered by employing the Polarizable Continuum Model (PCM) for water as the implicit solvent method, and the solvation model density (SMD) approach was applied.^76^^,^^77^ Hexane was considered as a model molecule of the polymer chain, and the abstraction of the H from carbon 3 was considered as the initial degradation step.

### Quantification and statistical analysis

All the results were the average of tri-measurements with standard error.

## Supplemental information

Document S1. Figures S1–S4 and Tables S1–S4

Table S5. Cartesian coordinates for the molecules used in the calculations, related to Figure 8

## References

[1] Mu Y., Sun J., Li Z., Zhang W., Liu Z., Li C., Peng C., Cui G., Shao H., Du Z. (2022). Activation of pyroptosis and ferroptosis is involved in the hepatotoxicity induced by polystyrene microplastics in mice. Chemosphere.

[2] Tanaka K., Takada H. (2016). Microplastic fragments and microbeads in digestive tracts of planktivorous fish from urban coastal waters. Sci. Rep..

[3] Naik R.A., Rowles L.S., Hossain A.I., Yen M., Aldossary R.M., Apul O.G., Conkle J., Saleh N.B. (2020). Microplastic particle versus fiber generation during photo-transformation in simulated seawater. Sci. Total Environ..

[4] Eriksen M., Lebreton L.C.M., Carson H.S., Thiel M., Moore C.J., Borerro J.C., Galgani F., Ryan P.G., Reisser J. (2014). Plastic Pollution in the World’s Oceans: More than 5 Trillion Plastic Pieces Weighing over 250,000 Tons Afloat at Sea. PLoS One.

[5] Schwarz A.E., Ligthart T.N., Boukris E., van Harmelen T. (2019). Sources, transport, and accumulation of different types of plastic litter in aquatic environments: A review study. Mar. Pollut. Bull..

[6] Barnes D.K.A., Galgani F., Thompson R.C., Barlaz M. (2009). Accumulation and fragmentation of plastic debris in global environments. Philos. Trans. R. Soc. Lond. B Biol. Sci..

[7] Kanomi S., Marubayashi H., Miyata T., Jinnai H. (2023). Reassessing chain tilt in the lamellar crystals of polyethylene. Nat. Commun..

[8] Miller M.S. (2020). Mapping Earth’s deepest secrets. Science.

[9] Elizalde-Velázquez G.A., Gómez-Oliván L.M. (2021). Microplastics in aquatic environments: A review on occurrence, distribution, toxic effects, and implications for human health. Sci. Total Environ..

[10] Prokić M.D., Radovanović T.B., Gavrić J.P., Faggio C. (2019). Ecotoxicological effects of microplastics: Examination of biomarkers, current state and future perspectives. TrAC, Trends Anal. Chem..

[11] Lau W.W.Y., Shiran Y., Bailey R.M., Cook E., Stuchtey M.R., Koskella J., Velis C.A., Godfrey L., Boucher J., Murphy M.B. (2020). Evaluating scenarios toward zero plastic pollution. Science.

[12] Fackelmann G., Pham C.K., Rodríguez Y., Mallory M.L., Provencher J.F., Baak J.E., Sommer S. (2023). Current levels of microplastic pollution impact wild seabird gut microbiomes. Nat. Ecol. Evol..

[13] Schirinzi G.F., Pérez-Pomeda I., Sanchís J., Rossini C., Farré M., Barceló D. (2017). Cytotoxic effects of commonly used nanomaterials and microplastics on cerebral and epithelial human cells. Environ. Res..

[14] Vethaak D., Legler J. (2021). Microplastics and human health. Science.

[15] Denisse Vital-Grappin A., Ariza-Tarazona M.C., Montserrat Luna-Hernández V., Francisco Villarreal-Chiu J., Manuel Hernández-López J., Siligardi C., Cedillo-González E.I., Mx J.M.H. (2021). The Role of the Reactive Species Involved in the Photocatalytic Degradation of HDPE Microplastics Using C,N-TiO 2 Powders. Polymers.

[16] Liu L., Xu M., Ye Y., Zhang B. (2022). On the degradation of (micro)plastics: Degradation methods, influencing factors, environmental impacts. Sci. Total Environ..

[17] Kim S., Seo A.Y., Lee T.G. (2020). Functionalized cellulose to remove surfactants from cosmetic products in wastewater. Carbohydr. Polym..

[18] Parvulescu V.I., Epron F., Garcia H., Granger P. (2022). Recent Progress and Prospects in Catalytic Water Treatment. Chem. Rev..

[19] Amelia D., Fathul Karamah E., Mahardika M., Syafri E., Mavinkere Rangappa S., Siengchin S., Asrofi M. (2022). Effect of advanced oxidation process for chemical structure changes of polyethylene microplastics. Mater. Today Proc..

[20] Rajput H., Kwon E.E., Younis S.A., Weon S., Jeon T.H., Choi W., Kim K.H. (2021). Photoelectrocatalysis as a high-efficiency platform for pulping wastewater treatment and energy production. Chem. Eng. J..

[21] Collivignarelli M.C., Abbà A., Carnevale Miino M., Arab H., Bestetti M., Franz S. (2020). Decolorization and biodegradability of a real pharmaceutical wastewater treated by H2O2-assisted photoelectrocatalysis on TiO2 meshes. J. Hazard Mater..

[22] Barham J.P., Chemie B.A. (2020). Synthetic Photoelectrochemistry. Angew. Chem. Int. Ed..

[23] Alulema-Pullupaxi P., Fernández L., Debut A., Santacruz C.P., Villacis W., Fierro C., Espinoza-Montero P.J. (2021). Photoelectrocatalytic degradation of glyphosate on titanium dioxide synthesized by sol-gel/spin-coating on boron doped diamond (TiO2/BDD) as a photoanode. Chemosphere.

[24] Qian R., Zong H., Schneider J., Zhou G., Zhao T., Li Y., Yang J., Bahnemann D.W., Pan J.H. (2019). Charge carrier trapping, recombination and transfer during TiO2 photocatalysis: An overview. Catal. Today.

[25] Rengifo-Herrera J.A., Pulgarin C. (2023). Why five decades of massive research on heterogeneous photocatalysis, especially on TiO2, has not yet driven to water disinfection and detoxification applications? Critical review of drawbacks and challenges. Chem. Eng. J..

[26] Zarei E., Ojani R. (2017). Fundamentals and some applications of photoelectrocatalysis and effective factors on its efficiency: a review. J. Solid State Electrochem..

[27] Oliveira T.M., Ribeiro F.W., Morais S., de Lima-Neto P., Correia A.N. (2022). Removal and sensing of emerging pollutants released from (micro)plastic degradation: Strategies based on boron-doped diamond electrodes. Curr. Opin. Electrochem..

[28] Pointer Malpass G.R., de Jesus Motheo A. (2021). Recent advances on the use of active anodes in environmental electrochemistry. Curr. Opin. Electrochem..

[29] Terashima C., Hishinuma R., Roy N., Sugiyama Y., Latthe S.S., Nakata K., Kondo T., Yuasa M., Fujishima A. (2016). Charge Separation in TiO2/BDD Heterojunction Thin Film for Enhanced Photoelectrochemical Performance. ACS Appl. Mater. Interfaces.

[30] Castillo-Cabrera G.X., Pliego-Cerdán C.I., Méndez E., Espinoza-Montero P.J. (2023). Step-by-step guide for electrochemical generation of highly oxidizing reactive species on BDD for beginners. Front. Chem..

[31] Castillo-Cabrera G.X., Espinoza-Montero P.J. (2024). Novel trends in mixed oxide electrodes for photoelectrocatalytic wastewater treatment. Curr. Opin. Electrochem..

[32] Huang J., Meng A., Zhang Z., Ma G., Long Y., Li X., Han P., He B. (2022). Porous BiVO4/Boron-Doped Diamond Heterojunction Photoanode with Enhanced Photoelectrochemical Activity. Molecules.

[33] Yao Y., Sang D., Duan S., Wang Q., Liu C. (2021). Review on the Properties of Boron-Doped Diamond and One-Dimensional-Metal-Oxide Based P-N Heterojunction. Molecules.

[34] Qu J., Zhao X. (2008). Design of BDD-TiO2 hybrid electrode with P-N function for photoelectroatalytic degradation of organic contaminants. Environ. Sci. Technol..

[35] Bastug Azer B., Gulsaran A., Pennings J.R., Saritas R., Kocer S., Bennett J.L., Devdas Abhang Y., Pope M.A., Abdel-Rahman E., Yavuz M. (2022). A Review: TiO2 based photoelectrocatalytic chemical oxygen demand sensors and their usage in industrial applications. J. Electroanal. Chem..

[36] Adak D., Chakrabarty P., Majumdar P., Mukherjee R., Patra S., Mondal A., Bhattacharyya S., Saha H., Bhattacharyya R. (2020). Pd nanoparticle-decorated hydrogen plasma-treated TiO2 for photoelectrocatalysis-based solar energy devices. ACS Appl. Electron. Mater..

[37] Sigcha-Pallo C., Peralta-Hernández J.M., Alulema-Pullupaxi P., Carrera P., Fernández L., Pozo P., Espinoza-Montero P.J. (2022). Photoelectrocatalytic degradation of diclofenac with a boron-doped diamond electrode modified with titanium dioxide as a photoanode. Environ. Res..

[38] An Y., Hou J., Liu Z., Peng B. (2014). Enhanced solid-phase photocatalytic degradation of polyethylene by TiO2-MWCNTs nanocomposites. Mater. Chem. Phys..

[39] Zhang K., Zhang K., Ma Y., Wang H., Shao J., Li M., Shao G., Fan B., Lu H., Xu H. (2023). Construction of Z-Scheme TiO2/Au/BDD Electrodes for an Enhanced Electrocatalytic Performance. Materials.

[40] Holder C.F., Schaak R.E. (2019). Tutorial on Powder X-ray Diffraction for Characterizing Nanoscale Materials. ACS Nano.

[41] Espinola-Portilla F., Navarro-Mendoza R., Gutiérrez-Granados S., Morales-Muñoz U., Brillas-Coso E., Peralta-Hernández J.M. (2017). A simple process for the deposition of TiO2 onto BDD by electrophoresis and its application to the photoelectrocatalysis of Acid Blue 80 dye. J. Electroanal. Chem..

[42] Xu J., Yokota Y., Wong R.A., Kim Y., Einaga Y. (2020). Unusual Electrochemical Properties of Low-Doped Boron-Doped Diamond Electrodes Containing sp2 Carbon. J. Am. Chem. Soc..

[43] Patel K., Hashimoto K., Fujishima A. (1992). Photoelectrochemical investigations on boron-doped chemically vapour-deposited diamond electrodes. J. Photochem. Photobiol. Chem..

[44] Green S.J., Mahe L.S.A., Rosseinsky D.R., Winlove C.P. (2013). Potential and ph dependence of photocurrent transients for boron-doped diamond electrodes in aqueous electrolyte. Electrochim. Acta.

[45] Pelskov Y., Sakharova A., Krotova M.D., Bouilov L.L., Spitsyn B.V. (1987). Photoelectrochemical properties of semiconductor diamond. J. Electroanal. Chem. Interfacial Electrochem..

[46] Djonse Justin B.T., Blaise N., Valery H.G. (2023). Investigation of the photoactivation effect of TiO2 onto carbon-clay paste electrode by cyclic voltammetry analysis. Heliyon.

[47] Youssef L., Roualdès S., Bassil J., Zakhour M., Rouessac V., Lamy C., Nakhl M. (2019). Effect of plasma power on the semiconducting behavior of low-frequency PECVD TiO2 and nitrogen-doped TiO2 anodic thin coatings: photo-electrochemical studies in a single compartment cell for hydrogen generation by solar water splitting. J. Appl. Electrochem..

[48] Chen P., Mu Y., Chen Y., Tian L., Jiang X.H., Zou J.P., Luo S.L. (2022). Shifts of surface-bound ^·^OH to homogeneous ^·^OH in BDD electrochemical system via UV irradiation for enhanced degradation of hydrophilic aromatic compounds. Chemosphere.

[49] Lu J., Hou R., Wang Y., Zhou L., Yuan Y. (2022). Surfactant-sodium dodecyl sulfate enhanced degradation of polystyrene microplastics with an energy-saving electrochemical advanced oxidation process (EAOP) strategy. Water Res..

[50] Medeiros de Araújo D., Cañizares P., Martínez-Huitle C.A., Rodrigo M.A. (2014). Electrochemical conversion/combustion of a model organic pollutant on BDD anode: Role of sp3/sp2 ratio. Electrochem. Commun..

[51] Wang M., Simon N., Decorse-Pascanut C., Bouttemy M., Etcheberry A., Li M., Boukherroub R., Szunerits S. (2009). Comparison of the chemical composition of boron-doped diamond surfaces upon different oxidation processes. Electrochim. Acta.

[52] Kiendrebeogo M., Karimi Estahbanati M.R., Khosravanipour Mostafazadeh A., Drogui P., Tyagi R.D. (2021). Treatment of microplastics in water by anodic oxidation: A case study for polystyrene. Environ. Pollut..

[53] Jiang R., Lu G., Yan Z., Liu J., Wu D., Wang Y. (2021). Microplastic degradation by hydroxy-rich bismuth oxychloride. J. Hazard Mater..

[54] Orimolade B.O., Arotiba O.A. (2020). Towards visible light driven photoelectrocatalysis for water treatment: Application of a FTO/BiVO4/Ag2S heterojunction anode for the removal of emerging pharmaceutical pollutants. Sci. Rep..

[55] Orimolade B.O., Arotiba O.A. (2022). Enhanced photoelectrocatalytic degradation of diclofenac sodium using a system of Ag-BiVO4/BiOI anode and Ag-BiOI cathode. Sci. Rep..

[56] Gelderman K., Lee L., Donne S.W. (2007). Flat-Band Potential of a Semiconductor: W Using the Mott-Schottky Equation. J. Chem. Educ..

[57] Han M., Jia J. (2016). 3D Bi2S3/TiO2 cross-linked heterostructure: An efficient strategy to improve charge transport and separation for high photoelectrochemical performance. J. Power Sources.

[58] Chen P., Mu Y., Chen Y., Tian L., Jiang X.H., Zou J.P., Luo S.L. (2022). Shifts of surface-bound ^·^OH to homogeneous ^·^OH in BDD electrochemical system via UV irradiation for enhanced degradation of hydrophilic aromatic compounds. Chemosphere.

[59] Jian Z., Yang N., Vogel M., Zhou Z., Zhao G., Kienitz P., Schulte A., Schönherr H., Jiao T., Zhang W., Jiang X. (2020). Tunable Photo-Electrochemistry of Patterned TiO2/BDD Heterojunctions. Small Methods.

[60] Makuła P., Pacia M., Macyk W. (2018). How To Correctly Determine the Band Gap Energy of Modified Semiconductor Photocatalysts Based on UV-Vis Spectra. J Phys Chem Lett.

[61] Krehula L.K., Katančić Z., Siročić A.P., Hrnjak-Murgić Z. (2014). Weathering of high-density polyethylene-wood plastic composites. J. Wood Chem. Technol..

[62] Bredács M., Barretta C., Castillon L.F., Frank A., Oreski G., Pinter G., Gergely S. (2021). Prediction of polyethylene density from FTIR and Raman spectroscopy using multivariate data analysis. Polym. Test..

[63] Almond J., Sugumaar P., Wenzel M.N., Hill G., Wallis C. (2020). Determination of the carbonyl index of polyethylene and polypropylene using specified area under band methodology with ATR-FTIR spectroscopy. E-Polymers.

[64] Campanale C., Savino I., Massarelli C., Uricchio V.F. (2023). Fourier Transform Infrared Spectroscopy to Assess the Degree of Alteration of Artificially Aged and Environmentally Weathered Microplastics. Polymers.

[65] Sorolla-Rosario D., Llorca-Porcel J., Pérez-Martínez M., Lozano-Castelló D., Bueno-López A. (2022). Study of microplastics with semicrystalline and amorphous structure identification by TGA and DSC. J. Environ. Chem. Eng..

[66] Yu J., Wang P., Ni F., Cizdziel J., Wu D., Zhao Q., Zhou Y. (2019). Characterization of microplastics in environment by thermal gravimetric analysis coupled with Fourier transform infrared spectroscopy. Mar. Pollut. Bull..

[67] Nabi I., Bacha A.U.R., Li K., Cheng H., Wang T., Liu Y., Ajmal S., Yang Y., Feng Y., Zhang L. (2020). Complete Photocatalytic Mineralization of Microplastic on TiO2 Nanoparticle Film. iScience.

[68] Hong Y., Oh J., Lee I., Fan C., Pan S.Y., Jang M., Park Y.K., Kim H. (2021). Total-organic-carbon-based quantitative estimation of microplastics in sewage. Chem. Eng. J..

[69] Brosler P., Girão A.V., Silva R.F., Tedim J., Oliveira F.J. (2023). In-house vs. commercial boron-doped diamond electrodes for electrochemical degradation of water pollutants: A critical review. Front. Mater..

[70] Zhang X., Li J., Fan W.Y., Sheng G.P. (2019). Photomineralization of Effluent Organic Phosphorus to Orthophosphate under Simulated Light Illumination. Environ. Sci. Technol..

[71] Kiendrebeogo M., Karimi Estahbanati M.R., Ouarda Y., Drogui P., Tyagi R.D. (2022). Electrochemical degradation of nanoplastics in water: Analysis of the role of reactive oxygen species. Sci. Total Environ..

[72] Ochoa-Chavez A.S., Pieczyńska A., Fiszka Borzyszkowska A., Espinoza-Montero P.J., Siedlecka E.M. (2018). Electrochemical degradation of 5-FU using a flow reactor with BDD electrode: Comparison of two electrochemical systems. Chemosphere.

[73] Brea O., Loroño M., Marquez E., Mora J.R., Cordova T., Chuchani G. (2012). Theoretical study of methoxy group influence in the gas-phase elimination kinetics of methoxyalkyl chlorides. Int. J. Quant. Chem..

[74] Cuesta S.A., Mora J.R., Meneses L.M., Márquez E.A., Flores-Morales V., Rincón L., Torres F.J., Zambrano C.H. (2022). Unveiling the structure-reactivity relationship involved in the reaction mechanism of the HCl-catalyzed alkyl t-butyl ethers thermal decomposition. A computational study. Int. J. Quant. Chem..

[75] Chen Z., Li Y., He Z., Xu Y., Yu W. (2019). Theoretical investigations on charge transport properties of tetrabenzo[a,d,j,m]coronene derivatives using different density functional theory functionals (B3LYP, M06-2X, and wB97XD). J. Chem. Res..

[76] Marenich A.V., Cramer C.J., Truhlar D.G., Donald G. (2009). Universal Solvation Model Based on Solute Electron Density and on a Continuum Model of the Solvent Defined by the Bulk Dielectric Constant and Atomic Surface Tensions. J. Phys. Chem. B.

[77] Marenich A.V., Cramer C.J., Truhlar D.G., Donald G. (2009). Performance of SM6, SM8, and SMD on the SAMPL1 Test Set for the Prediction of Small-Molecule Solvation Free Energies. J. Phys. Chem. B.

