# Supplementary material for: Photoelectrocatalytic degradation of high-density polyethylene microplastics on TiO2-modified boron-doped diamond photoanode
Source: iScience. 2024 Feb 12;27(3):109192. doi: 10.1016/j.isci.2024.109192 (PMC10906510; doi:10.1016/j.isci.2024.109192)
Supplement: Document S1. Figures S1–S4 and Tables S1–S4 [file mmc1.pdf]

iScience, Volume *27*

Supplemental information

Photoelectrocatalytic degradation of high-density polyethylene microplastics on TiO_2-modified boron-doped diamond photoanode

Wendy Quilumbaquin, G. Xavier Castillo-Cabrera, Luis J. Borrero-González, José R. Mora, Vladimir Valle, Alexis Debut, Luis D. Loor-Urgilés, and Patricio J. Espinoza-Montero

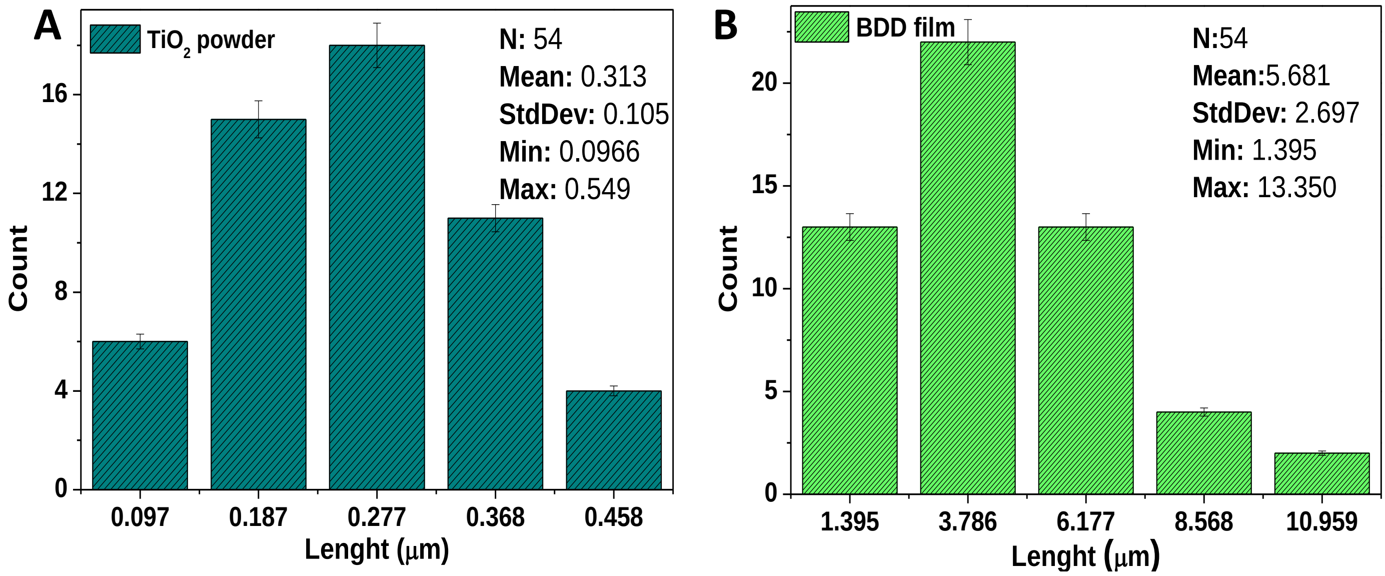

Figure S1. Particle size by SEM analysis, Related to Figure 1.

(A) TiO_2 powder.

(B) BDD film.

Data are represented as mean ± SEM.

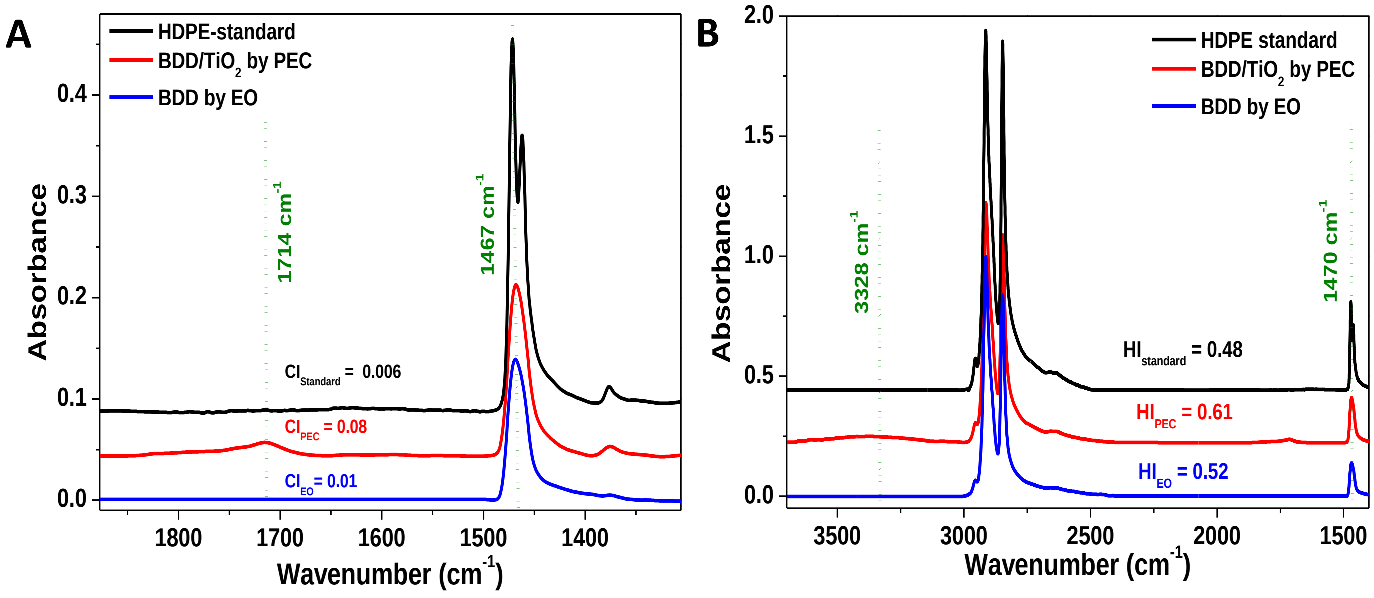

Figure S₂. FTIR spectra of HDPE-MPs before and after 10 h reaction by EO using bare BDD and PEC using BDD/TiO2 photoanode, Related to Figure 4.

(A) Carbonyl Index

(B) Hydroxyl Index

Gravimetric analysis

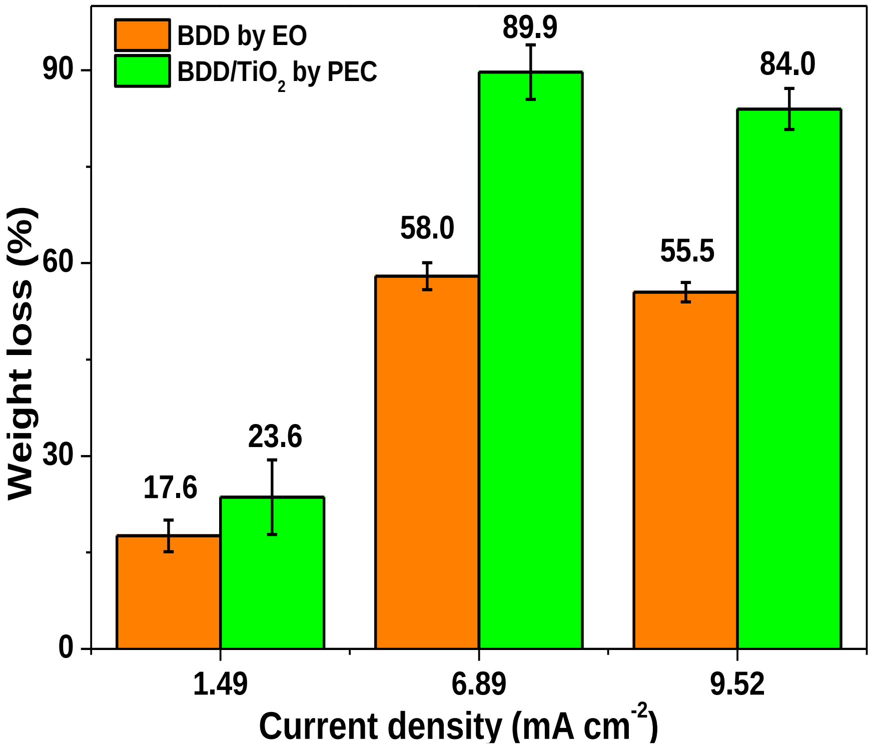

Figure S3. Gravimetric analysis GA) in HDPE-MPs, Related to Figure 4.
Data are represented as mean ± SEM.

Thermogravimetric analysis

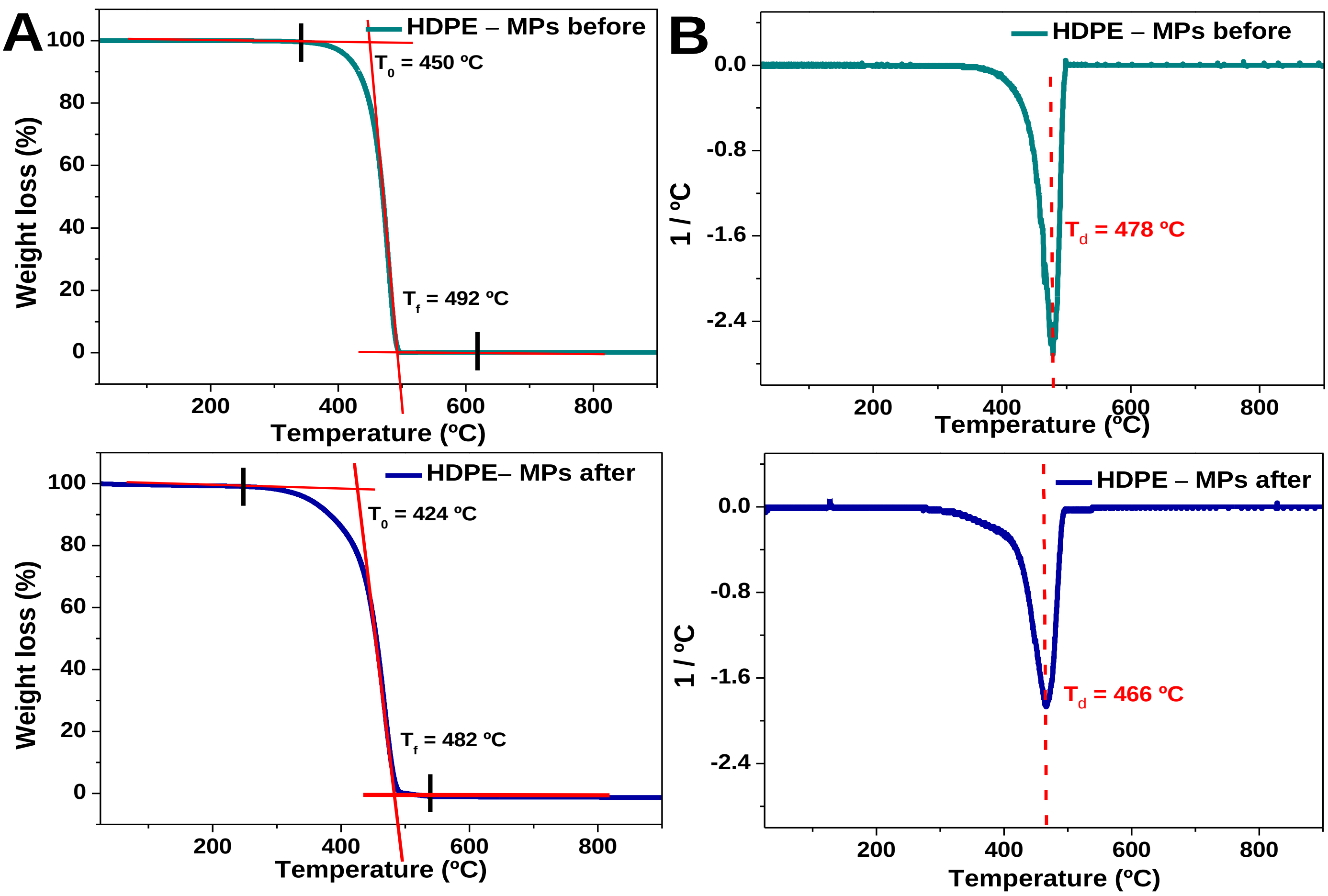

Figure S4. Thermogravimetric analysis (TGA) in HDPE-MPs, Related to Figure 4

(A) TGA plots of HDPE-MPs before (top) and after (bottom) PEC treatment. The temperature range used was 25-900°C with the heating rate of 10°C·min^{-1}.

(B) First derivate of thermogravimetric analysis of HDPE-MPs before (top) and after (bottom) PEC treatment.

Table S1. Electroactive area calculous of BDD and BDD/TiO_2 photoanode in darkness and under UV-light, Related to Figure 2.

Conditions	Electroactive area (mA cm^{-2})	Kinetic charge transfers constant k° (cm s^{-1})
**BDD[a]**	6.29	3.15×10^{-4}
**BDD under UV-light**	8.35	3.97×10^{-4}
**BDD/TiO_2**	10.78	4.65×10^{-4}
**BDD/TiO_2 under UV-light**	13.59	5.42×10^{-4}

[a]BDD (Boron doped diamond) and UV-light (Ultraviolet light).

Table S2. Weight loss in EO and PEC after 10 h, Related to Figure 4.

	WEIGHT LOSS (%)	
Current density (mA·cm^{-2})	Electrochemical oxidation (EO)	Photoelectrocatalysis (PEC)
1.49	17.58 ± 0.03	23.58± 0.05
6.89	57.97 ± 0.09	89.91± 0.08
9.52	55.48± 0.12	83.98± 0.07

Table S3. The onset, end-set and peak values of the HDPE-MPs' thermal degradation, Related to Figure 4.

	WEIGHT THERMOGRAVIMETRYC ANALYSIS (10°C·min-1)			
**Conditions**	**Onset**	**End-Set**	**Peak Temperature**	
	Temperature (°C)	Temperature (°C)	Temperature (°C)	
**HDPE-MPs[a] before**	450.42	492.06	478.88	28.498
**HDPE-MPs after**	424.11	482.86	466.33	30.333

[a]HDPE-MPs (High density polyethylene microplastic).

Table S4. TOC and COD analysis in EO and PEC after 10 h, Related to Figure 6.

	Electrochemical oxidation (EO)		Photoelectrocatalysis (PEC)	
Current density (mA·cm^{-2})	TOC[a] (mg L^{-1} O_2)	COD[b] (mg L^{-1} O_2)	TOC (mg L^{-1} O_2)	COD (mg L^{-1} O_2)
1.49	3.6	9	10.5	26.5
6.89	250	633	396.2	1003
9.52	11.1	28.2	1.7	4.2

[a]TOC (Total Organic Carbon), [b]COD (Chemical Oxygen Demand).
